# Characterization of the Relationship between the Loess Moisture and Image Grayscale Value

**DOI:** 10.3390/s21237983

**Published:** 2021-11-30

**Authors:** Qingbing Liu, Jinge Wang, Hongwei Zheng, Tie Hu, Jie Zheng

**Affiliations:** 1 Three Gorges Research Center for Geohazards of the Ministry of Education, China University of Geosciences, Wuhan 430074, China; qingbing@cug.edu.cn (Q.L.); zhenghwcug@sina.com (H.Z.); hutie@cug.edu.cn (T.H.); 2 School of Civil, Environmental & Mining Engineering, The University of Western Australia, Crawley 6009, Australia; 3 Faculty of Information Engineering, Wuhan University of Engineering Science, Wuhan 430200, China; zhengjiewh@hotmail.com

**Keywords:** loess, water content, digital images, grayscale value, mathematical model

## Abstract

This paper presents a model for estimating the moisture of loess from an image grayscale value. A series of well-controlled air-dry tests were performed on saturated Malan loess, and the moisture content of the loess sample during the desiccation process was automatically recorded while the soil images were continually captured using a photogrammetric device equipped with a CMOS image sensor. By converting the red, green, and blue (RGB) image into a grayscale one, the relationship between the water content and grayscale value, referred to as the water content–gray value characteristic curve (WGCC), was obtained; the impacts of dry density, particle size distribution, and illuminance on WGCC were investigated. It is shown that the grayscale value increases as the water content decreases; based on the rate of increase of grayscale value, the WGCC can be segmented into three stages: slow-rise, rapid-rise, and asymptotically stable stages. The influences that dry density and particle size distribution have on WGCC are dependent on light reflection and transmission, and this dependence is closely related to soil water types and their relative proportion. Besides, the WGCC for a given soil sample is unique if normalized with illuminance. The mechanism behind the three stages of WGCC is discussed in terms of visible light reflection. A mathematical model was proposed to describe WGCC, and the physical meaning of the model parameters was interpreted. The proposed model is validated independently using another six different types of loess samples and is shown to match well the experimental data. The results of this study can provide a reference for the development of non-contact soil moisture monitoring methods as well as relevant sensors and instruments.

## 1. Introduction

Loess is a yellowish, predominantly silt-sized sediment that is rich in calcium carbonate and most abundant in semi-arid regions of inner Eurasia [1]. Loess and loess-like deposits are one of the most widespread Quaternary sedimentary formations; the Loess Plateau of China has the thickest and most complete loess strata in the world [2,3,4]. Loess is well known for its high water sensitivity, which is strongly related to its mineralogy and meta-stable microstructure. Within the theoretical framework of unsaturated soil mechanics, it is recognized that the variation of moisture content in unsaturated loess leads to the redistribution of matric suction and suction stress, and hence is the key factor affecting the strength and deformation behaviors such as collapsibility [5,6,7,8,9,10,11]. The increase in soil moisture content due to rainfall and other hydrologic processes is the main cause that account for many geotechnical problems and disasters in loess areas, such as ground subsidence and collapse, abrupt and excessive settlement of foundation, and landslides, leading to great economic losses and casualties [12,13,14]. Therefore, the rapid and accurate assessment of the moisture content of loess is of importance to engineering construction practice in loess-covered regions.

The current techniques for soil moisture measurement can be classified into direct, indirect, and remote sensing methods. The oven-drying method is a direct, accurate way to measure the moisture content by weighing the sample before and after drying. Indirect methods use an instrument or sensor placed in the soil to measure some soil properties related to the moisture and can be divided into two categories: radiological techniques, such as neutron scattering and gamma absorption, and dielectrics methods, such as time domain reflectometry, frequency domain measurement, and amplitude domain reflectometry [15,16,17]. These indirect approaches can provide rapid and continuous measurement; however, they require careful and sophisticated calibration procedures for inferring soil moisture [18,19]. Remote sensing technology can serve as a non-contact method to monitor soil moisture on a large scale. Multispectral, hyperspectral, thermal infrared, and microwave remote sensing measurement of soil moisture have been widely applied in agriculture-related fields [20,21,22,23]. The principle of the remote sensing method is based on the physical relationship between soil moisture content and a specific reflectance spectrum. The surface soil moisture content, on a regional scale, is obtained by the interpretation of the remotely sensed data. Existing studies show that remote sensing techniques are capable of well depicting seasonal and short-term soil moisture changes. However, biases in the absolute value and dynamic range may be large when compared with in situ measured and modeled soil moisture data [24,25].

Drawing upon the principle of the remote sensing technique, the image-based methods have been proposed and increasingly applied to measure soil moisture content [26,27,28,29,30,31,32,33].The image-based technique commonly employs an ordinary digital camera to capture soil sample, and the moisture content is derived from soil color using the pre-established calibration curve. It is obvious that the calibration relationship between soil moisture content and its color is the key for employing the image-based technique. In this regard, Persson [26] used four natural soils and uniform fine sand to investigate the variation of soil color with moisture content in both the red, green, and blue (RGB) and hue, saturation, and value (HSV) color spaces, and proposed using a simple regression model between S and V in the HSV color space to estimate soil moisture. Hafizah and Khairunniza [27] showed that the CIELUV color space gave better image interpretation of soil moisture content compared to RGB and HSV. Zhu et al. [28] found that there was an exponential negative correlation between the soil surface gray level (SGL) and the moisture content. Zhang et al. [29] proposed an online method for detecting soil surface moisture using image processing techniques and an approximate calibration relationship between the moisture and image grayscale value. Rahimi-Ajdadi et al. [30] developed a machine vision model for predicting the soil moisture using image analysis; this model enables an estimate of soil moisture by only using three color features, including the mode of the color channel of blue and means of the color channels of normalized red and blue. Kim et al. [31] developed a water content estimation equation using soil color and suggested a calibration method for field application of this equation to predict soil water content with unmanned aerial vehicle (UAV) images. Besides, Kirillova et al. [32] investigated calibration methods for measuring the color of moist soils with digital cameras and showed that a decrease in soil moisture leads to a significant increase in lightness and yellowness.

Overall, classical oven-drying or gravimetric method is point-based, destructive, and unable to provide continuous measurement of soil moisture while indirect methods (e.g., radiological and dielectrics techniques) have the disadvantages of small-scale measurement, low accuracy, limited spatial coverage, high preliminary cost, and accuracy dependent on the quality of employed sensors, sophisticated calibration procedure, and in situ setup of instruments. The remote sensing technique for estimating soil moisture is suitable for large areas having uniform coverage; however, it is considered as a complex and expensive technique, and its accuracy is restricted by uncertainties associated with the lighting, temperature, interruption by clouds and foliage, and considerable signal interruption by the Earth’s atmosphere. Compared with the above-mentioned methods, the image-based method for soil moisture estimation is considered as promising because it has the advantages of being practical, fast, non-destructive, relatively low cost, and enables real-time continuous measurement in the field [33]. Nevertheless, there are few studies on the application of the image analysis method to evaluate loess moisture, and no calibration relationship between soil color and moisture content has ever been reported for loess. Therefore, the aim of this study is to characterize the relationship between the loess moisture and relevant image parameters, and the concept of “water content–grayscale value characteristic curve (WGCC)” is proposed. On this basis, a mathematical model of WGCC is established for estimating loess moisture from an image grayscale value. The proposed WGCC model can be expected to improve the applicability and accuracy of the image-based method for estimating or monitoring loess moisture content.

## 2. Test Materials

### 2.1. Loess Soil

**Figure 1:**
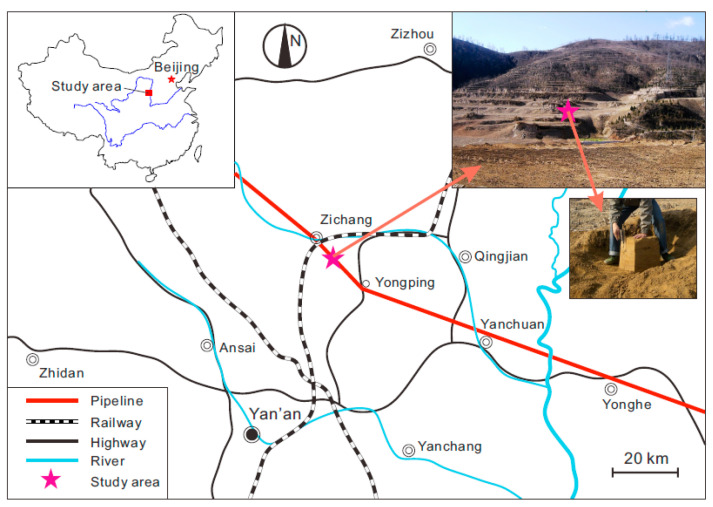
Location and photos of sampling area.

**Figure 2:**
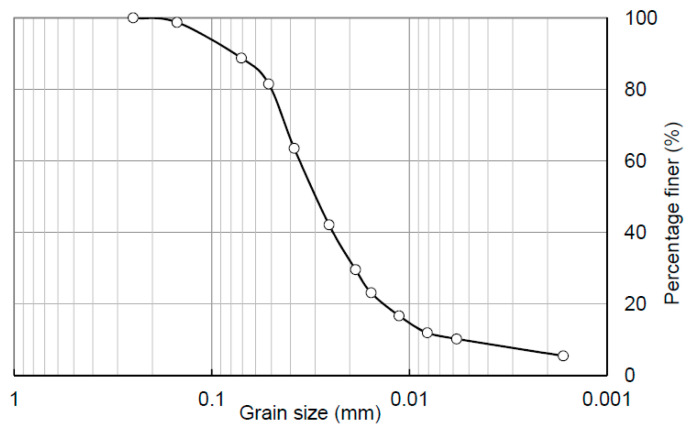
Particle size distribution curve of intact loess sample.

China’s West-to-East Gas Pipeline Project is a national key project, aiming to transport the natural gas reserves from the Tarim basin in the Xinjiang Autonomous Region in far western China to markets in eastern China. The pipeline has a total length of over 4000 km and crosses a large area of the Loess Plateau of China. The water-induced geo-hazards in loess areas posed a significant threat to the safe operation of this pipeline project [34,35,36,37]. To assess and mitigate the risk of pipeline failure caused by water-induced loess disaster, a series of research programs supported by the Chinese government has been carried out, which include the present study. The soil used in this study is typical Malan loess collected from the region between Zichang County and the town of Yongping in Shanxi Province, where the West-to-East Gas Pipeline passes through (Figure 1). The intact loess was cut into cubes with a dimension of 0.5 m × 0.5 m ×0.5 m, and then sealed and shipped back to the laboratory. Table 1 provides the basic properties of the tested loess while Figure 2 shows the particle size distribution curve.

### 2.2. Sample Preparation

Loess is a typical silt soil, and its water retention characteristic is controlled by the pore structure which significantly influences the distribution of soil pore water, and hence the relationship between the water content and soil color. As the dry density and particle size distribution are two critical factors affecting the pore structure or fabric of loess, these two indices are considered when designing the experimental scheme. Besides, the illumination intensity is the most crucial external factor that affects the soil imaging and hence the WGCC. For the purpose of separately examining the influences of dry density, particle-size distribution, and illumination, three individual groups of soil specimens were prepared; each group contained four specimens that have equally spaced values of the investigated parameter with the other two parameters held constant. As indicated in Table 2, the specimens D1~D4 have varying dry densities while the specimens G1~G4 are with different particle size distributions. These eight specimens were tested at the same illuminance of 5000 lux, whereas the specimens L1~L4, four identical specimens, were tested at the illuminance varying from 5000 to 7000 lux. It needs to be pointed out that the values of dry density and particle size distribution index employed for experiments fall into the normal range of loess in the studied area, and that the employed illuminance values are typical of light conditions controlled in the laboratory studies of the image analysis method.

**Figure 3:**
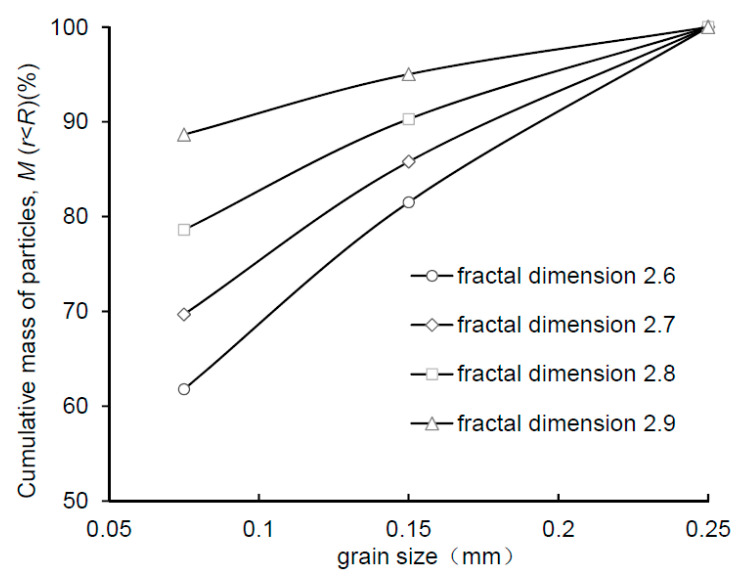
Fractal dimension of particle mass size distribution for artificial specimen G1~G4.

Considering the fractal characteristics of soil particle size distribution of natural loess [38,39,40], the mass fractal dimension *D* is used as a target indicator when preparing specimens with different particle size distribution. The mass fractal dimension *D* is calculated using the following equation:

(1) $$\frac{M\left(r<R\right)}{M_{T}}={\left(\frac{R}{R_{L}}\right)}^{3-D}$$

where *M* (*r* < *R*) represents the cumulative mass of particles with a size below an upper limit *R*; *M_T_* is the total mass of soil particles; *R_L_* is the upper size limit for fractal scaling; *D* is the mass fractal dimension or termed fractal dimension of particle mass size distribution. The cumulative mass of particles (*M* (*r* < *R*)) versus the particle size (*R*) for the specimens G1~G4 are plotted in Figure 3. For preparing a specimen, the predetermined mass of air-dried soil is weighed and then placed into a cylinder container, where the soil is compacted to the desired dry density. All of the specimens have a diameter of 61.8 mm and a height of 5 mm and are fully saturated using the vacuum saturation method prior to the testing.

## 3. Test Apparatus and Methods

### 3.1. Test Instruments and Procedures

**Figure 4:**
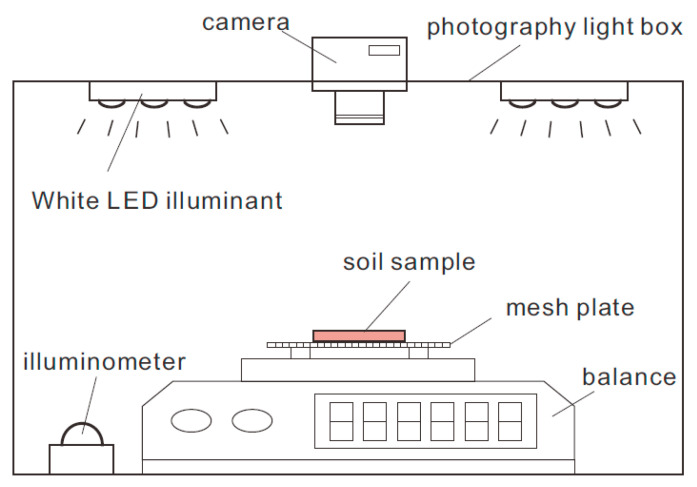
Schematic view of air-dry test setup.

The experiments were carried out using a self-made apparatus shown in Figure 4. The device consists of four main components: a photography light box, a digital camera, a digital electronic scale, and the illumination equipment. The light box has a cubic shape with a side length of 40 cm. The digital camera mounted on the top center of the light box is equipped with a 1/2.5-inch CMOS sensor and has a resolution of 14 million pixels. The electronic scale has a maximum weight capacity of 200 g with the resolution of 0.01 g and can output real-time measurements through the RS485 interface. During the air-dry test, the soil specimen is put on a steel wire mesh plate that is placed beforehand on the weighing pan of the electronic scale so that the top and bottom surfaces of the sample are in full contact with the atmosphere, thereby ensuring the homogeneous drying of the sample. Besides, two white LED light sources with adjustable illuminance were installed on the top of the light box and an illuminometer was used to monitor the illuminance within the box in real time.

The test process includes the following steps: (1) place presaturated loess sample on the steel wire mesh plate and record its weight, and then seal the light box; (2) adjust the illuminance within the box to the desired value and focus the digital camera to the top surface of the loess sample; (3) the time interval for capturing soil image and recording weight is set to 10 min, and the test is stopped when the reading of electronic scale changes by less than 0.02 g within 4 h; (4) dry the sample in an oven at a temperature of 105 °C for 24 h, and then weigh the sample for determining the moisture content.

### 3.2. Data Processing

The RGB images of the loess samples obtained with the digital camera were first converted into grayscale images. The RGB-to-grayscale conversion methods are mainly divided into two types. One basic approach is to use the single channel, such as the red, green, or blue color component of RGB color space. However, such an approach may lead to loss of discriminative information due to exploiting only one image channel. The other approach that is commonly used and widely adaptive is generating a grayscale image by equally contributing the *R*, *G*, and *B* color channels, as described by Equation (2) below.

(2) $$G=(R_{ed}+G_{reen}+B_{lue})/3$$

where *G* is the grayscale value ranging from 0 to 255; *R_ed_*, *G_reen_* and *B_lue_* are the intensity values of the red, green, and blue channels for each pixel in an RGB image. The larger the *G* value is, the closer the image is to pure white or totally bright. Herein, the Equation (2) is adopted to convert the RGB images of loess specimens.

The obtained grayscale images were then further processed using the mean filtering method in order to smooth the images by reducing undesirable image noise that can cause error of experimental results. The image noise is produced by the image sensor and circuitry of the employed digital camera. Another important reason for using the mean filtering technique is that the loess contains a few dark-colored minerals with low reflectivity, leading to dark or black dots. These dark dots could induce the obvious decrease of grayscale value in local areas of images and hence the underestimation of the water content. Therefore, they are considered as noisy data and need to be eliminated through mean filtering performed on the grayscale image. The idea of mean filtering is to replace each pixel value in an image with the mean (or average) value of its neighbors, including itself, as illustrated in the following formula.

(3) G(x,y)=∑f (x,y)/m

where *G*(*x*, *y*) is the grayscale value of a pixel with the pixel coordinate of (*x*, *y*) in a processed image; *f*(*x*, *y*) is the grayscale value of a neighboring pixel and *m* is the total number of neighboring pixels involved in the calculation.

**Figure 5:**
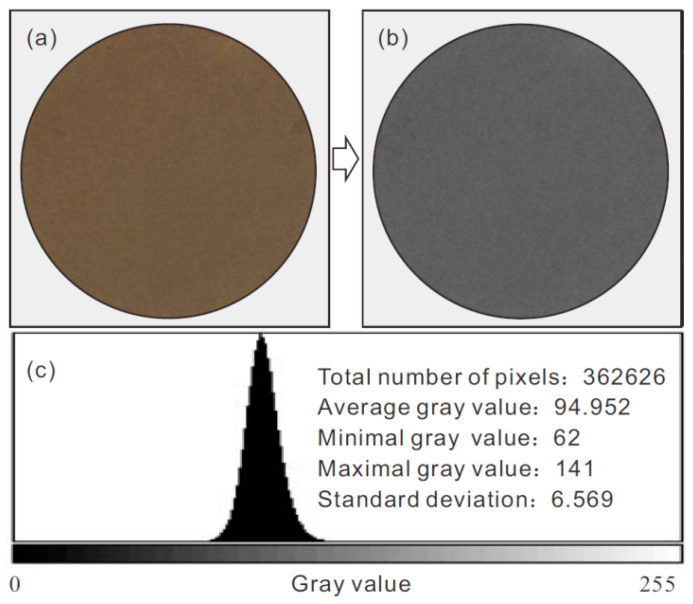
Conversion of color image to grayscale image and grayscale histogram: (**a**) original image of sample; (**b**) grayscale image and (**c**) image histogram representing the grayscale value distribution.

Figure 5a,b shows an RGB image of a loess sample that is converted to grayscale mode and subjected to mean filtering. Figure 5c presents an example image histogram representing the grayscale value distribution (i.e., the frequency of occurrence of each gray-level value). The statistical analysis indicates that the grayscale values of all of the pixels in a grayscale image approximately follow the normal distribution. Herein, the average grayscale value of all of the pixels of an image is taken as the representative grayscale value of this image. In this way, the representative grayscale values of the soil image captured at some point during the air-dry test can be determined, which is then plotted against the corresponding water content to obtain the water content–grayscale value characteristic curve (WGCC).

## 4. Results

### 4.1. Effect of Dry Density on WGCC

**Figure 6:**
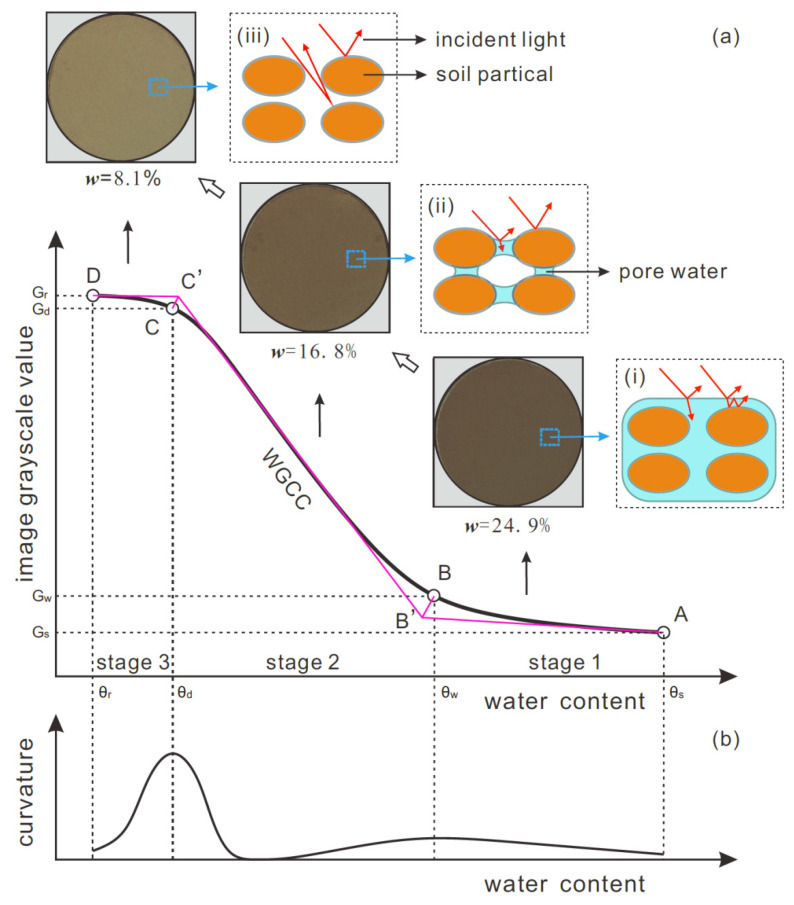
Typical WGCC: (**a**) graphic method to derive inflection points and intrinsic correlation of WGCC stage transition with soil water type; (**b**) WGCC curvature and the analytical method for determining inflection points; (i), (ii) and (iii) are light reflection and refraction in stage 1, 2 and 3, respectively.

It is found that the obtained WGCCs for all specimens are inverse S-shaped and that the grayscale value increases as the water content decreases. A typical WGCC is presented in Figure 6, where the light reflection status at each stage of WGCC is schematically illustrated. Based on the rate of increase of the grayscale value, the WGCC can be segmented into three stages: slow-rise (stage 1), rapid-rise (stage 2), and asymptotically stable stages (stage 3). To facilitate the following interpretations, four feature points of the WGCC are defined here, namely, saturation point, air-dry endpoint, wet inflection point, and dry inflection point. These four points are illustrated as point A, B, C, and D in Figure 6a, respectively. The saturated point corresponds to the initially saturated state of the loess specimen with a (saturated) volumetric moisture content of *θ_s_* and a grayscale value of *G*_s_. The air-dry endpoint refers to the final air-dried state of the specimen with a residual volumetric moisture content of *θ_r_* and a grayscale value of *G_r_*. The wet inflection point is the point at which WGCC changes from the slow-rise to the rapid-rise stage while the dry inflection point refers to the point at which WGCC turns from the rapid-rise stage to the asymptotically stable stage. 

As indicated in Figure 6a, the dry and wet inflection points can be derived using a graphical method. This method involves three steps: (1) drawing a tangent line to the WGCC at the saturation point, air-dry endpoint, and middle point of the rapid-rise stage, respectively, and the lines intersect at B’ and C’; (2) plotting the bisector of the angle between tangent lines passing the saturation point and the middle point of the rapid-rise stage as well as the bisector of the angle between tangent lines passing the air-dry endpoint and the middle point of the rapid-rise stage; (3) the points of intersection of two angle bisectors with WGCC are considered as wet and dry inflection points. Clearly, these two inflection points indicate the sudden change in the rate of increase of the grayscale value with decreased water content, and their physical implications will be discussed later in Section 5.1. The volumetric water content and grayscale value of the soil specimen at wet and dry inflection points are denoted as *θ_w_*, *G_w_*, and *θ_d_*, *G_d_*, respectively.

**Figure 7:**
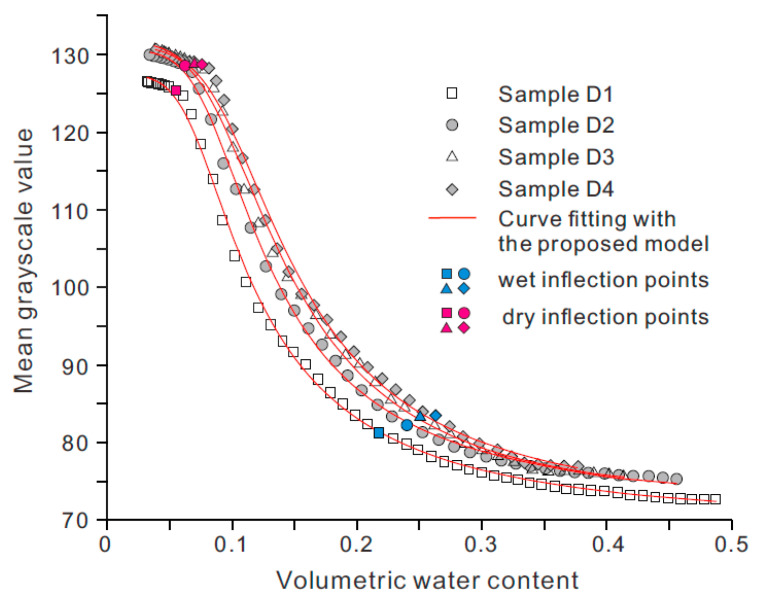
WGCCs for samples with different dry densities.

Figure 7 shows the WGCCs of loess samples with different dry densities. it is seen that as the dry density increases, the WGCC tends to shift towards the positive direction of the horizontal and vertical axes, and an increase in dry density leads to the decrease of *θ_s_* and the increase of *θ_r_*, *θ_w_*, and *θ_d_*. As *θ_s_* is positively related to soil porosity and four specimens have the same bulk volume, an increase in dry density causes the porosity and *θ_s_* to decrease. The residual volumetric water content *θ_r_* is related to the amount of water tightly absorbed on soil particles. Thus, the specimen with a higher dry density contains more soil particles retaining tightly absorbed water, resulting in the increase of *θ_r_*.

The increase of *θ_w_* with dry density indicates that the slow-rise stage of WGCC is shorter for the sample with higher dry density, whereas the increase of *θ_d_* with dry density implies a longer asymptotically stable stage for the sample of higher dry density. Additionally, it is seen that at a given volumetric water content, the grayscale value increases with dry density. This can be explained by the denser fabric and lower surface roughness of the sample with high density, which reduces the light transmission and improves the reflection of light from the sample surface leading to an increase in the grayscale value of the soil image.

### 4.2. Effect of Particle Size Distribution on WGCC

**Figure 8:**
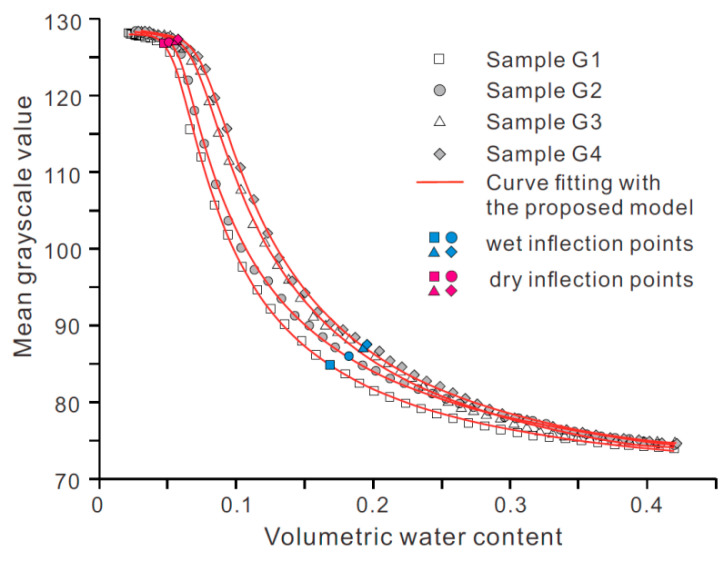
WGCCs for samples with different particle size distributions.

Figure 8 shows WGCCs of specimens G1~G4 with varying fractal dimension of particle mass size distribution. The two ends (i.e., saturation point and air-dry endpoints) of four curves are seen to approximately coincide, whereas the dry and wet inflection points of them differ from each other. Generally, *θ_w_* and *θ_d_* increase as the mass fractal dimension increases. Specimens G1~G4 have an identical initial bulk volume and dry density, so the specimen with higher mass fractal dimension implies that it contains less coarse particles and more fine particles. In this sense, the increase of *θ_w_* and *θ_d_* with the mass fractal dimension indicates that a higher fraction of fine particles can induce a shorter slow-rise stage of WGCC and a longer asymptotically stable stage. Besides, Figure 8 shows that, at a given volumetric water content, the grayscale value of the soil image increases with mass fractal dimension. This may arise from the decrease of the sample surface roughness owing to more fine particles filling the void space between coarse particles, which leads to the enhancement of light reflection.

### 4.3. Influence of Illumination on WGCC

**Figure 9:**
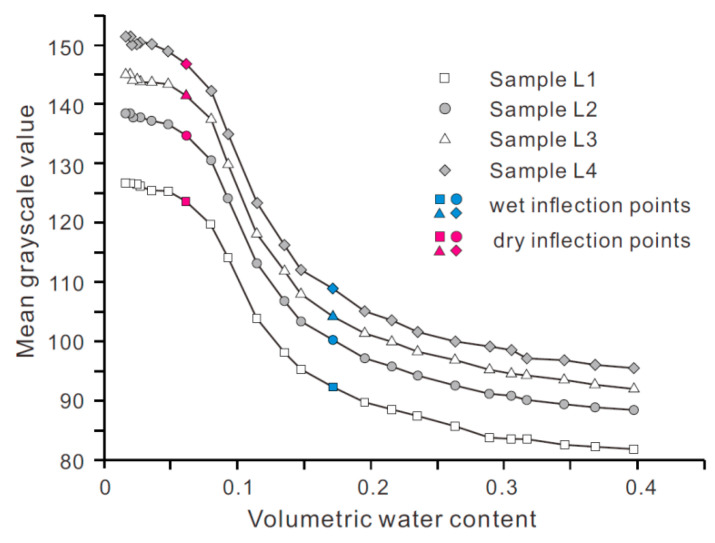
WGCCs for samples tested at different levels of illuminance.

Light condition is a key external factor controlling the quality of the soil image captured, thus affecting the accuracy of the water content estimation from the image analysis. Figure 9 presents WGCCs of four specimens tested at an illumination intensity ranging from 5000 to 7000 lux.

The most striking observation made from the figure is that four curves are nearly parallel to each other along the *Y*-axis direction. Furthermore, at a given water content, the grayscale value is almost linearly proportional to the illuminance. These observations have an important implication that when the camera parameters remain unchanged, there is a unique relationship between the grayscale value normalized with illuminance and the volumetric water content. In other words, WGCC obtained at any illuminance can be converted to the one measured under a reference illumination condition by shifting it a distance determined using the pre-established linear relationship between illuminance and grayscale value. This finding, to some extent, demonstrates that the use of the grayscale value of a soil image to interpret the moisture content has good adaptability to changing circumstances.

## 5. Discussion

### 5.1. Intrinsic Correlation between Soil Water Type and WGCC Stage Transition

In the slow-rise stage (stage 1) bound by the saturation point and wet inflection point, the soil specimen has a high water content with a relatively high degree of saturation, and the pore space of soil is filled with a large amount of free (gravitational) water. In this case, as illustrated in Figure 6a-(i), the light striking the soil top surface is partly reflected off the surface water film and received by the camera while the rest is refracted into the soil–water system where it undergoes multiple refraction–reflection and a high energy dissipation, resulting in only a small amount of light escaping from the soil–water system and being received by the camera. As a consequence, the soil images captured in this stage are relatively dark with a low grayscale value. Besides, the duration of the slow-rise stage of WGCC is highly related to the amount of free water in soil pores in view of the fact that the soil water lost in this stage is mainly the free water. As previously mentioned, the soil specimen in this stage has a high degree of saturation; as the water on soil top surface evaporates, the free water in soil pores is continuously transported upward, which maintains the evaporation front close to the near-surface of the soil specimen. This causes a slow process for the soil particles to emerge from the water or to be exposed to the air, which is manifested by a relatively slow increase in the grayscale value with decreasing water content. For the same reason, WGCCs for the specimens with a low dry density or mass fractal dimension show a longer slow-rise stage (Figure 8 and Figure 9) due to the fact that they have more total pore space or higher macroporosity to accommodate free water.

The rapid-rise stage (stage 2) of WGCC is bound by wet and dry inflection points indicated in Figure 6a. As the water content approaches the wet inflection point, a vast majority of free water has evaporated, and the remaining soil water is mainly capillary water. As illustrated in Figure 6a-(ii), the soil pores are filled with air and capillary water that bridges the soil particles. In this case, when a beam of light is incident on the soil surface, most of the light is reflected off the soil surface particles and then received by the camera, which is accompanied by a small amount of light refracted into the pore capillary water and then subjected to multiple refraction–reflection and energy dissipation. Thus, the brightness of the soil images captured during this stage is enhanced and the grayscale value is increased when compared with stage 1. Besides, capillary water has low interconnectivity, and the decrease of capillary water leads to a greater curvature of air–water capillary meniscus between particles. Therefore, the soil particles are quickly exposed to air during this stage of evaporation, resulting in a rapid increase in the grayscale value of the soil image.

With a further decrease of water content, the capillary water completely evaporates and the WGCC steps into the third stage, as indicated in Figure 6a-(iii). During this stage, the soil contains only bound water that tightly covers the soil particles in the form of a thin water film. The evaporation rate in this stage is very low as the bound water is bonded with soil particles with high energy, and the light incident the soil surface can be fully reflected and received by the camera with very little light being refracted. In this situation, the obtained soil images show much higher brightness with a very high grayscale value. Due to the fact that the amount of bound water in soil is rather small and that most of the soil particles have been exposed to air, the grayscale value of soil images shows a marginal variation and finally reaches a quasi-steady state.

### 5.2. WGCC Model

It is found that the experimentally derived WGCCs of the employed Malan loess can be described using a unified model below.

(4) $$G\left(\theta \right)=K\times \left[G_{s}+\frac{G_{r}-G_{s}}{{\left(1+a\times \theta^{b}\right)}^{c}}\right]$$

where *G*(*θ*) is the image grayscale value of the soil specimen with volumetric water content of *θ*; *G_s_* is the image grayscale value of the soil specimen under initially saturated sate (i.e., saturation point); *G_r_* is the image grayscale value of the soil specimen under finally air-dried state (i.e., air-dry endpoint); *a*, *b*, and *c* are model parameters while *K* is illuminance-related correction factor. Clearly, after being calibrated, the model (Equation (4) can be used for estimating the water content from the grayscale value of a loess image. It is of note that when the illuminance of the in situ measurement is identical to that employed in the calibration process, the correction factor *K* is not considered and takes the value of 1. Otherwise, if the illuminance used in practice differs from that of the calibration process, the correction factor *K* needs to be estimated by establishing the linear relationship between the illuminance and grayscale value at a given water content (i.e., *K* equals to the linear slope). With the calibrated factor *K*, the grayscale value obtained under various in situ illuminances can be normalized to the grayscale value under any specified reference illuminance, thereby making the result unique.

Based on the proposed model, the wet and dry inflection points can be determined using an analytical method which defines two inflection points as the points where the curvature of WGCC reaches peak values. The WGCC curvature κ can be calculated with the following Equation (5), and the variation of WGCC curvature with moisture content is illustrated in Figure 6b.

(5) $$\kappa =\frac{\left|\left(G/100\right)’’\right|}{{\left(1+\left(G/100\right){’}^{2}\right)}^{3/2}}$$

where *κ* is the curvature; *G* equals *G*(*θ*) of Equation (4), which is normalized by 100 to uniform the scales of the horizontal and vertical axes; *G*’ and *G*” are the first and second derivative of Equation (4) with respect to *θ*. It is clear that this analytical method and the graphical method described in Section 4.1 are based on a common principle: the two inflection points represent the sudden change in the rate of increase of the grayscale value with decreased water content. It is found that the inflection points obtained from the graphical method are in reasonable agreement with those determined using the analytical method.

The Equation (4) was used to model experimental data of loess specimens with varying dry density and particle size distribution, as illustrated in Figure 7 and Figure 8. The best-fit values of model parameters and R-squared values (*R*^2^) are provided in Table 3. The proposed model is found to match experimental data closely with all of the R^2^ values exceeding 0.99 (Table 3). For the two groups of tested loess specimens, the order of magnitude for the best-fit values of parameters *a*, *b,* and *c* ranges from 10^3^ to 10^5^, 1 to 10, and 0.1 to 1, respectively.

### 5.3. WGCC Model Parameters and Implications

**Figure 10:**
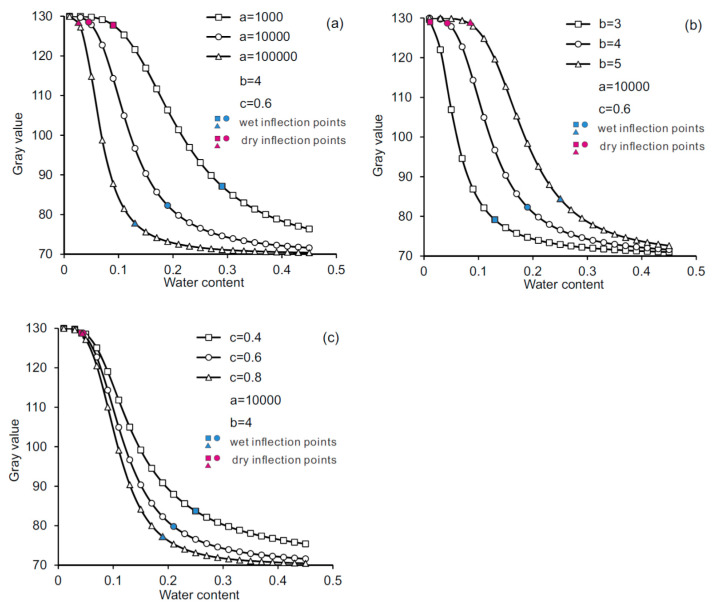
Comparisons of WGCCs using different model parameter values: (**a**) WGCCs with varying parameter *a*; (**b**) WGCCs with varying parameter *b* and (**c**) WGCCs with varying parameter *c*.

The implications of model parameters *a*, *b,* and *c* were investigated by examining the effects of their values on the generated WGCCs. The values for model parameters *a*, *b*, and *c* are varied within their respective ranges of the above-mentioned order of magnitude. It is of note that parameters *G_s_* and *G_r_* take the constant values of 70 and 130 to generate the WGCCs. Figure 10a presents three WGCCs obtained by varying parameter *a* value from 1000 to 100,000 and using the fixed values for parameter *b* and *c*. It is seen that as *a* value increases, both the dry and wet inflection points shift to the left and the dry inflection point moves a greater distance than the wet inflection point, which implies that the duration of stage 1 increases while the duration of the stages 2 and 3 decreases. Based on the intrinsic linkage between soil water type and WGCC stage transition elaborated in Section 5.1, it can be inferred that parameter *a* is related to the amount of free water in the soil. A higher *a* value indicates that the soil specimen has more large pores and higher free water content with a relatively small amount of capillary water and bound water. The influence of parameter *b* on WGCC is illustrated in Figure 10b, where an increase in *b* value is seen to cause the dry and wet inflection points to move approximately the same distance to the right, indicating the decrease of the duration of stage 1, increase of the duration of stage 3, and nearly unchanged duration of stage 2. Based on the interpretations made in Section 5.1, the effect of *b* value on stage 1 and stage 3 implies that this parameter is correlated with the fine particle content of loess. A higher *b* value indicates that the soil specimen has a higher content of fine particles that can carry more bound water. Figure 10c shows that the effect of parameter *c* on the wet inflection point is similar to that of parameter *a*. However, the variation of *c* value exerts no influence on the dry inflection point and the duration of stage 3. An increase in *c* value induces increased duration of stage 1 and decreased duration of stage 2, implying that parameter *c* is linked to the relative proportion of free water and capillary water in a loess sample.

### 5.4. Validation of WGCC Model

**Figure 11:**
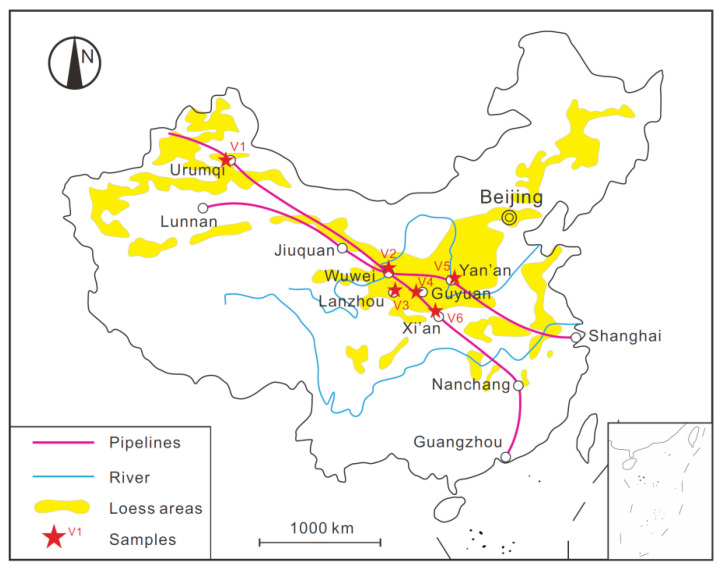
Sampling locations of six types of loess used for validating the WGCC model.

Considering that the WGCC model presented in Equation (4) is established by using experimental data of only one type of Malan loess, more loess samples are collected from the field and then tested to validate the proposed model. As illustrated in Figure 11, these samples are retrieved from six different loess regions where the West-to-East Gas Pipeline passes through and their detailed information is provided in Table 4. It is seen from the Table 4 that, apart from the difference in the sampling location, the six loess samples (V1 to V6) vary in in situ dry density, chronological age, and granulometric composition, and thus, basically, can represent a variety of loess types distributed along the pipeline project.

Unlike the reconstituted loess specimens used in the previous testing program (Table 2), the validation experiments employed undisturbed specimens. Six different loess samples with the intact structure were carefully trimmed to the required specimen shape and then fully saturated. All of the specimens were tested at the same illuminance of 5000 lux following the procedures described in Section 3.1.

**Figure 12:**
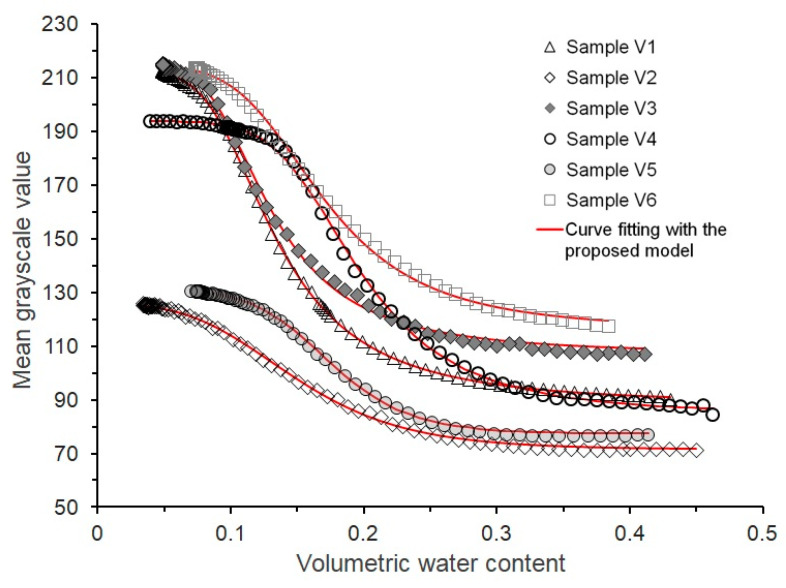
WGCCs of six loess samples collected from different regions.

The obtained WGCCs of the six loess samples are presented in Figure 12, where the adequacy and fitness of Equation (4) to describe these WGCCs are also examined. The results of model fitting and the best-fit parameter values are listed in Table 5. It is clear that although the WGCCs of the six loess samples differ significantly, the proposed model can fit the experimental data of each sample very well with all of the R-squared values (R^2^) exceeding 0.99, thereby indicating that it has a wide applicability for different types of loess soils. Besides, Figure 12 indicates that different types of loess may possess a distinct range of grayscale values between the saturated and air-dried states; however, this distinction has no effect on the suitability of the Equation (4) for characterizing the general relationship between loess moisture and an image grayscale value. In other words, for any given loess, the proposed model is capable of reasonably estimating or predicting its moisture content from the image grayscale value after being well calibrated.

## 6. Conclusions

This paper investigated the relationship between the water content of loess and image grayscale value, referred to as water content–grayscale value characteristic curve (WGCC). It is shown that WGCCs of tested loess specimens are inverse S-shaped curves and can be divided into three distinct stages delimited by four feature points: saturation point, dry inflection point, wet inflection point, and air-dry endpoint. The dry density and particle size distribution significantly influence WGCC as they control the pore structure and water retention behavior of the soil, which are key factors affecting the light reflection and refraction on the air–soil interface. It is found that there is a unique relationship between the grayscale value normalized with illuminance and volumetric water content. The mechanism behind the three distinct stages of WGCC can be explained by a different light reflection/refraction status at the three phases of the soil desiccation process, which correspond to the evaporation of free water, capillary water, and bound water, respectively. A simple WGCC model (Equation (4)) is proposed, which is validated independently by another six different loess samples and is shown to match all of the experimental data well. Besides, the implications of model parameters are discussed in terms of their correlation with soil water type.

The results presented in this study have the potential to be applied in practice for estimating the loess moisture both on the laboratory scale and in a field investigation. For example, for the laboratory centrifuge or 1-g (normal gravity) model test, the ordinary or micro digital camera can be mounted to photograph the physical model of loess (e.g., loess subgrade, embankment) that is subjected to a drought or rainfall climate simulation. Then, the moisture content profile can be estimated based on the analysis of the captured images using the proposed method; the obtained data on moisture fluctuation can support the interpretation of the relevant geotechnical issue in a loess area (e.g., slope failure, collapsible foundation, ground fissure). For the field applications, the proposed method allows for dynamically monitoring the moisture of a surface loess deposit within a small area by analyzing the images captured by a drone; it can also be applied to evaluate the moisture distribution along any loess profile excavated in slope, foundation, tunnel, and pile engineering. Besides, it can be envisaged that the development of a new non-contact instrument or sensor for soil moisture measurement is possible, as long as an image acquisition device is packaged with a software module that can automatically analyze image parameters and invoke an implanted calibration relationship (e.g., the proposed model) to output moisture content.

## Figures and Tables

**Table 1:** Basic properties and mineral composition of studied loess.

Basic Properties	Values	Mineral	Content (%)
In situ water content *ω*	20.8%	Quartz	40
In situ density, *ρ*	1.46 g/cm^3^	Feldspar	15
Liquid limit, *w_L_*	32.2%	Calcite	10
Plastic limit, *w_p_*	20.2%	Clay minerals	30
Specific gravity, *G_s_*	2.71	Others	5

**Table 2:** Initial parameters of tested loess specimens.

Specimen ID	Dry Density (g/cm^3^)	Mass Fractal Dimension	Illumination (lux)
D1	1.4	2.62	5000
D2	1.5	2.62	5000
D3	1.6	2.62	5000
D4	1.7	2.62	5000
G1	1.6	2.6	5000
G2	1.6	2.7	5000
G3	1.6	2.8	5000
G4	1.6	2.9	5000
L1	1.6	2.6	5000
L2	1.6	2.6	6000
L3	1.6	2.6	6500
L4	1.6	2.6	7000

**Table 3:** The result of model fitting and parameters.

Specimen ID		D1	D2	D3	D4	G1	G2	G3	G4
Input values	*G*_s_	72.7	75.3	76.3	76.9	74.2	74.4	74.5	74.6
	*G*_r_	127.8	130.5	131.2	130.8	128.6	129.0	128.6	129.1
Best-fit values	*a*	6335	4347	3788	2813	94,620	84,070	17,310	48,470
	*b*	3.781	3.921	4.021	3.967	4.406	4.472	4.234	4.688
	*c*	0.634	0.741	0.754	0.819	0.479	0.448	0.576	0.499
R-squared	*R* ^2^	0.997	0.998	0.997	0.996	0.998	0.997	0.996	0.997

**Table 4:** Basic information of loess samples used for model validation.

Sample ID	V1	V2	V3	V4	V5	V6
Sampling location	Urumchi	Wuwei	Lanzhou	Guyuan	Yan’an	Xi’an
Chronological age	Q3	Q3	Q2	Q3	Q2	Q2
In situ dry density/g·cm^−3^	1.48	1.45	1.59	1.42	1.56	1.61
Clay fraction/% (<0.005 mm)	8.4	13.5	15.5	11.2	16.8	21.4
Silt fraction/% (0.075–0.005 mm)	82.8	81.9	82.0	85.3	79.4	75.9
Sand fraction/% (>0.075 mm)	8.8	4.6	2.5	3.5	3.8	2.7

Q2: middle Pleistocene; Q3: late Pleistocene.

**Table 5:** The results of model fitting to experimental data for six different loess samples.

Sample ID		V1	V2	V3	V4	V5	V6
Input values	*G_s_*	89.5	71.4	107.2	84.5	77.4	118.1
	*G_r_*	212.4	125.4	215.2	193.8	130.8	214.2
Best-fit values	*a*	110,626	154	214,960	62,373	4213	11,970
	*b*	5.287	3.063	5.551	6.353	5.147	5.020
	*c*	0.537	1.898	0.543	0.638	1.639	0.718
R-squared	*R* ^2^	0.997	0.999	0.996	0.998	0.998	0.997

## Data Availability

The data presented in this study are available on request from the corresponding author.
